# COPD heterogeneity: implications for management

**DOI:** 10.1186/s40248-016-0053-4

**Published:** 2016-03-17

**Authors:** Miriam Barrecheguren, Marc Miravitlles

**Affiliations:** Pneumology Department, Hospital Universitari Vall d’Hebron, P. Valld’Hebron 119-129, 08035 Barcelona, Spain

Chronic obstructive pulmonary disease (COPD) is a complex and heterogeneous disease and its definition as a disease characterised by a non completely reversible airflow obstruction mainly caused by cigarette smoke does not fully describe the variability in its clinical presentation.

The main complain of patients with COPD is exertional dyspnea caused by hyperinflation [1]. Therefore, the basis of treatment is the optimal bronchodilation, which is also effective in reducing the future risk of exacerbations [2]. However, despite optimal bronchodilation, some patients may experience episodes of exacerbation. In order to manage these more challenging patients we need to take into account the diversity of the disease. In recent years, there has been a growing interest in defining and characterising the different types of COPD patients, commonly known as “clinical phenotypes” [3]. These phenotypes include patients that share clinical features and similar response to available treatments, and may have prognostic implications [4].

Yet in 2012, the Spanish guidelines of COPD (GesEPOC) identified four different phenotypes with clinical relevance and therapeutic repercussion: the non exacerbator phenotype, the exacerbator phenotype with emphysema or with chronic bronchitis and the asthma-COPD overlap syndrome (ACOS) [5]. The exacerbator phenotype is defined as two or more exacerbations during the previous year [6]. The initial differentiation between exacerbators and non exacerbators is crucial to prevent the inadequate use of anti-inflammatory drugs in patients without exacerbations. Among the exacerbators, GesEPOC identifies three different phenotypes; frequent exacerbators with emphysema or with chronic bronchitis and ACOS, which can also present with frequent exacerbations [7].

Exacerbators with emphysema should receive long acting bronchodilators (LABD) combined with inhaled corticosteroids (ICS) when LABD are not enough to control symptoms and exacerbations, and in particular if they have high concentrations of eosinophils in peripheral blood [8]. They can also benefit from pulmonary rehabilitation and endoscopic volume reduction in carefully selected cases. For patients with frequent exacerbations and chronic bronchitis, there are other anti-inflammatories in addition to ICS that can provide benefits, such as roflumilast, mucolytics at high doses or long-term macrolides in very selected cases and under close supervision [5]. ACOS patients use to have increased airflow reversibility and eosinophil counts in sputum and peripheral blood [7]. Some studies demonstrated that patients with COPD and high eosinophil blood counts treated with ICS presented a significant reduction in exacerbations compared with patients treated with LABA alone [9]. Therefore, ACOS patients should be treated with a combination of LABA/ICS from early stages, irrespective of the number of exacerbations in order to improve symptoms and control eosinophilic inflammation. Frequently, ACOS patients use to be asthmatics who smoked and developed non-fully reversible airflow limitation. In fact, a previous diagnosis of asthma in a patient with COPD can strongly suggest the diagnosis of ACOS [10]. Furthermore, LABA/ICS will be the first line treatment in exacerbators that have evidence of increased eosinophilic inflammation. However, the debate about the magnitude of eosinophilic inflammation necessary to consider the use of ICS is still ongoing.

The phenotypic approach to treatment has been followed by other European guidelines such as the Czech Republic, the Finnish, the Russian or the Swedish ones [11]. Some of the previously described phenotypes, such as the exacerbator or the ACOS, are acknowledged by all, while the Czech guidelines also include the COPD-bronchiectasis and the pulmonary cachexia [12].

The identification of clinical phenotypes of COPD reflects the heterogeneity of the disease and helps clinicians to select the most suitable treatment for their patients. The phenotypes proposed in recent guidelines are easy to identify in routine clinical practice.
